# Two-Dimensional Phototransistors with van der Waals Superstructure Contacts for High-Performance Photosensing

**DOI:** 10.1021/acsami.4c16883

**Published:** 2025-01-13

**Authors:** Ming-Deng Siao, Meng-Yu Tsai, Ashish Chhaganlal Gandhi, Yi-Chung Wu, Ta Fan, Li-Syuan Hao, I-Ling Li, Sun-Zen Chen, Chang-Hua Liu, Yen-Fu Lin, Chao-Hui Yeh

**Affiliations:** † Department of Electrical Engineering, National Tsing Hua University, Hsinchu 30013, Taiwan; ‡ Institute of Electronics Engineering, National Tsing Hua University, Hsinchu 30013, Taiwan; § Center for Nanotechnology, Materials Science and Microsystem, National Tsing Hua University, Hsinchu 30013, Taiwan; ∥ Department of Physics, National Chung Hsing University, Taichung 40227, Taiwan; ⊥ College of Semiconductor Research, National Tsing Hua University, Hsinchu 30013, Taiwan

**Keywords:** transition metal dichalcogenides, 2D phototransistors, photodetection, alternating WS_2_−WSe_2_ strip superstructure, type-II staggered band alignment, optoelectronics

## Abstract

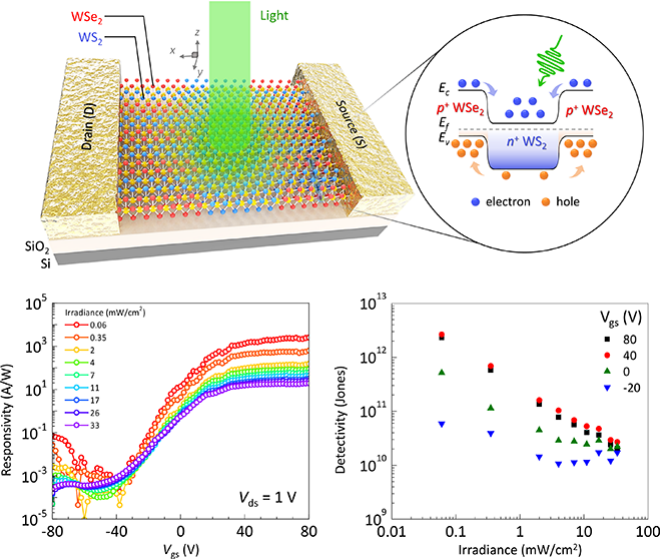

Semiconducting transition metal dichalcogenides (TMDs) possess exceptional photoelectronic properties, rendering them excellent channel materials for phototransistors and holding great promise for future optoelectronics. However, the attainment of high-performance photodetection has been impeded by challenges pertaining to electrical contact. To surmount this obstacle, we introduce a phototransistor architecture, in which the WS_2_ channel is connected with an alternating WS_2_–WSe_2_ strip superstructure, strategically positioned alongside the source and drain contact regions. Illumination triggers efficient separation of photoexcited electrons and holes due to the type-II staggered band alignment within the superstructure. Consequently, the contact regions exhibit degenerately doped n^+^ WS_2_ and p^+^ WSe_2_ strips under light illumination, resulting in minimal contact resistivity with the metal electrodes. The resultant WS_2_ phototransistor exhibits a remarkable responsivity of 2.4 × 10^6^ mA/W and an impressive detectivity of 2.6 × 10^12^ Jones. Furthermore, our time-resolved measurements reveal the absence of persistent photoconductance. This proposed phototransistor architecture provides a route for high-performance photodetection, effectively surpassing previous limitations associated with electrical contact.

## Introduction

In the field of photodetectors, efficient transformation of light energy into electrical signals is crucial, where photodiodes and phototransistors are the primary technologies^1,2^ used for this purpose. With respect to energy conversion, photodiodes competently separate photogenerated charge carriers through reverse bias-induced electric fields, resulting in substantial quantum efficiency.^3^ However, they require increased reverse bias and external signal amplification, limiting their integration with prevalent circuit architectures like complementary metal–oxide–semiconductor (CMOS).^4^ On the other hand, phototransistors, which consist of a transistor channel and an optical control gate, provide inherent amplification to photogenerated charge carriers, eliminating the need for complex external circuitry compared to photodiodes. This positions phototransistors as a promising alternative for photosensing applications, especially in scenarios demanding compatibility with commonly used architectures and simplified circuit design paradigms.^5,6^ Extensive research on photodetectors using low-dimensional materials such as zero-dimensional (0D) quantum dots, one-dimensional (1D) nanowires, and two-dimensional (2D) materials has provided valuable insights.^7−10^ The high photoresponse in 1D nanowires is attributed to their large surface-to-volume ratio and surface states, leading to surface Fermi-level pinning.^11,12^ However, they face challenges in returning to the initial dark state after light exposure due to surface band bending, resulting in prolonged carrier lifetimes and photoconductance insusceptible.^13^

Among 2D materials, transition metal dichalcogenide (TMD)-based phototransistors have shown promise, particularly due to their high in-plane carrier mobility and ultrathin thickness.^14,15^ While graphene photodetectors offer ultrafast response times, their responsivity is limited by a zero bandgap.^16,17^ TMDs, with their sizable bandgaps of 1–2 eV,^18^ allow for low-noise, high-sensitivity detection by switching the transistor channel to the off-state with an appropriate back-gate voltage, making TMD-based 2D phototransistors an effective solution for advanced photodetection applications.^19,20^ Recent studies on van der Waals heterostructure-based photodetectors have predominantly concentrated on vertical stacking. The weak van der Waals forces, in contrast to the strong in-plane covalent bonds, are appropriate for maintaining the vertical stack, resulting in fewer trap states within the interlayer of 2D heterostructures.^21^ The staggered band alignment in TMD heterostructures is particularly advantageous for photodetector channels, as it facilitates high photoresponse through ultrafast interlayer charge transfer between TMDs.^22,23^ For instance, Shin et al. reported a WSe_2_/MoS_2_ heterobilayer photodetector with impressive metrics, including a responsivity of 2700 A/W and a specific detectivity of 5 × 10^11^ Jones.^24^ Furthermore, hybrid photodetector channels formed from lateral TMD–TMD heterostructures also demonstrate high photoresponse and rapid response times.^25^ The unimpeded electrical transport of photon-excited carriers in TMD-based photodetectors is essential for achieving a high photoresponse.^26^ Typically, a Schottky barrier is present at the TMD/metal contact area due to the Fermi level pinning effect. However, graphene, with its gate-tunable carrier concentration and low-state density near the Dirac point, is suitable for contact engineering in TMD-based photodetectors.^27^ A notable example is the In/graphene–WS_2_–graphene hybrid photodetector, which achieved a high responsivity of 2600 A/W and ultrafast photoresponse.^28^ Tan et al. also reported a graphene-contacted WS_2_/MoS_2_ heterobilayer photodetector with a high responsivity of 2340 A/W and a gain of ∼5.4 × 10^3^, although its decay time was relatively prolonged.^29^

**Figure 1:**
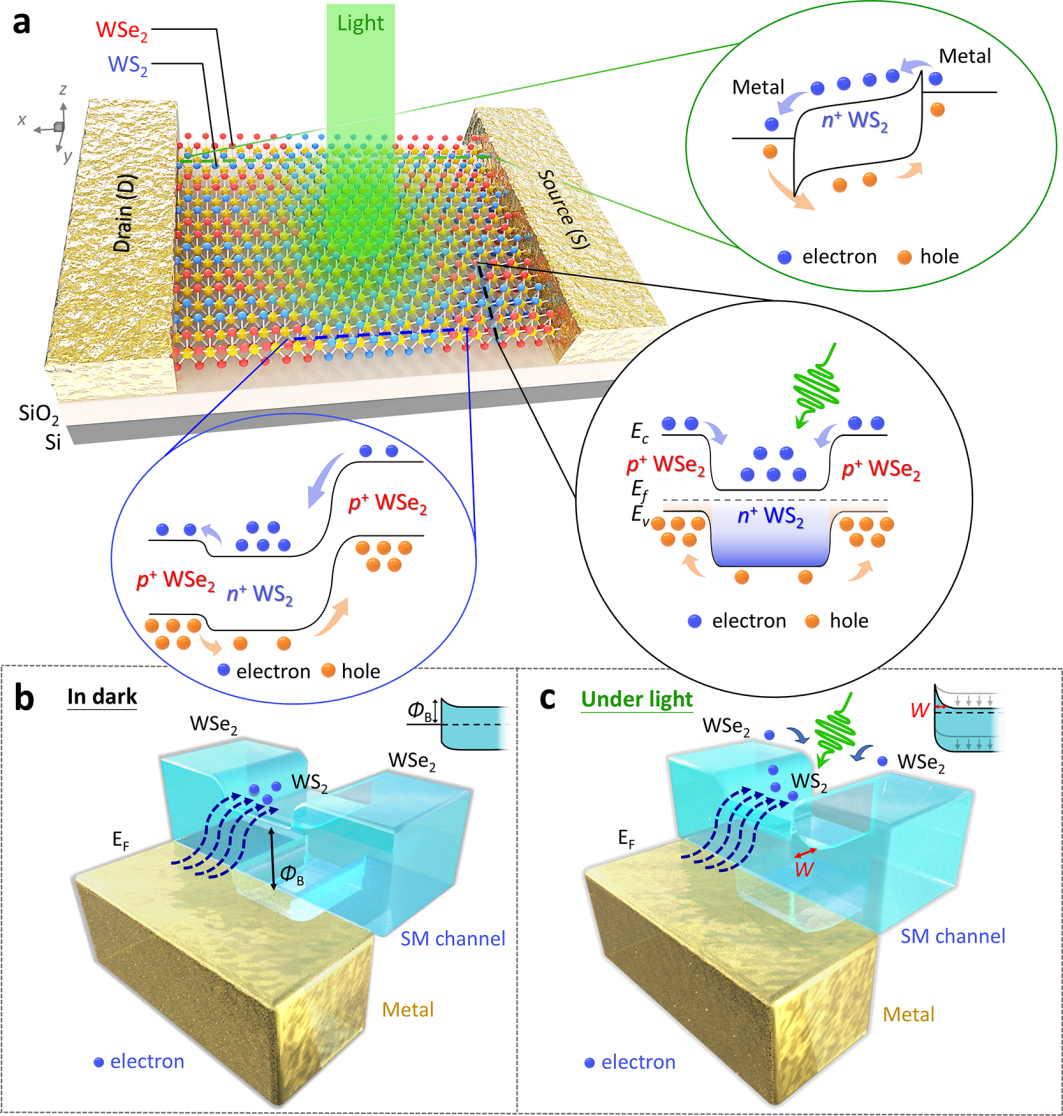
Schematic of a WS_2_–WSe_2_ SMS phototransistor with type-II superstructure. (a) Proposed SMS phototransistor with core WS_2_ channel and corresponding heterojunction band diagrams of transverse/longitudinal WS_2_–WSe_2_–WS_2_ as well as core WS_2_ configurations with the *V*_ds_ bias applied and presence of light. (b and c) Band diagrams of metal/WS_2_–WSe_2_ contacts with the absence and presence of light, respectively.

This study focuses on a novel high-performance phototransistor consisting of WS_2_ and WSe_2_ sub-micrometer strips (SMSs) as illustrated in Figure 1, where longitudinal SMS_∥_ (along the *x* axis) and transverse SMS_⊥_ (along the *y* axis) represent the arrangements of the strips. In particular, the transverse SMS_⊥_ points out the alternative WS_2_–WSe_2_ strips extending along the edges of the source and drain contacts (*y* axis), while SMS_∥_ features a longitudinal configuration of WSe_2_–WS_2_–WSe_2_ heterostructures along source to drain (*x* axis), sandwiched in parallel with homogeneous WS_2_ strips. Thus, it can be seen that the device gives prominence to a unique design where longitudinal WS_2_ strips form the core of n-type transistor channels integrated with WS_2_–WSe_2_ heterojunction lying on in-plane *x* direction. Through the channel, the corresponding band diagrams (SMS_⊥_ and SMS_∥_) shown in Figure 1a clarify the carrier transport in the heterojunctions and core WS_2_ as applied bias on the drain terminal.

The fabrication process for the WS_2_–WSe_2_ SMS phototransistor is clearly illustrated with optical microscopy (OM) images, as shown in Figure S1 of the Supporting Information. This arrangement results in a type-II structure that efficiently separates photoexcited electrons and holes within the heterostructure, leveraging the built-in potential at the heterojunction interfaces.^30^ The design choice of sub-micrometer-sized WS_2_ strips leads to a high concentration of photoexcited electrons in the channel, significantly enhancing electron transport. Additionally, light illumination reduces the contact barrier at the metal/WS_2_ interfaces, contributing to the device’s outstanding performance. These features culminate in the WS_2_–WSe_2_ SMS phototransistor achieving a high responsivity of 2.4 × 10^6^ mA/W and a detectivity of 2.6 × 10^12^ Jones, demonstrating the potential of WS_2_ and WSe_2_ SMSs as effective channel materials in advanced photodetection applications.

## Results and Discussion

**Figure 2:**
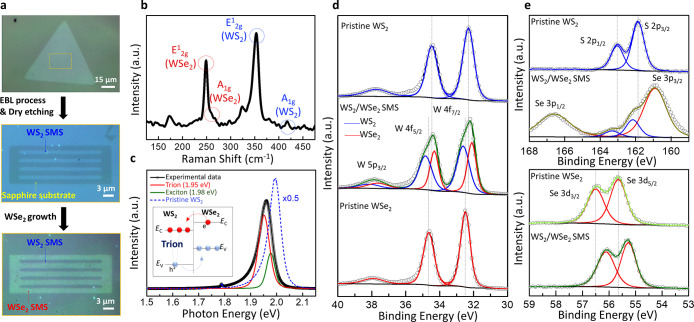
Optical characterization and XPS ore energy of semi-micro strip. (a) Optical images of WS_2_–WSe_2_ SMS processing steps from as-grown WS_2_ single crystal, well-patterned WS_2_ SMS, to heterostructure stitching using the two-step CVD approach. (b) Raman signatures of WS_2_–WSe_2_ SMS at the boundary interface. (c) Experimental photoluminescence of WS_2_–WSe_2_ SMS to fit exciton and trion curves of WS_2_. (d and e) Core-level binding energy of tungsten acquired by X-ray photoelectron spectroscopy. The binding energies of tungsten 4f_5/2_ and 4f_7/2_ in the WS_2_–WSe_2_ superstructure are compared to those in pristine WS_2_ and WSe_2_.

Figure 2a shows optical images of as-grown WS_2_–WSe_2_ SMS. The WS_2_ crystal serving as a canvas along with well-patterned strips achieved by the dry etching process can be a substantial template for sequential WSe_2_ growth to form WS_2_–WSe_2_ superlattice stitching.^31^ The specified orange dash located at the center of the WS_2_ crystal (Figure 2a) corresponds to the region for the following lateral WS_2_–WSe_2_ heterostructure stitching. The detailed Raman spectra in Figure 2b indicate the signature peaks of *E*_2g_^1^ of 248.5 cm^–1^/353.2 cm^–1^ and *A*_1g_ of 258.7 cm^–1^/417.2 cm^–1^ for WSe_2_ and WS_2_, respectively (Figure 2b).^32^ The photoluminescence (PL) spectra of the WSe_2_–WS_2_ heterostructure, when excited with a 532 nm laser source, reveal a broadened signature of the WS_2_ PL peak with two distinct peaks: a trion peak at 1.95 eV and an exciton peak at 1.98 eV, compared to the pristine WS_2_ PL peak at approximately 2.0 eV. This phenomenon can be explained by considering a couple of key factors: (I) The close proximity of the two different materials facilitates charge transfer between the layers. This charge transfer can lead to the formation of trions (charged excitons), which consist of either two electrons and one hole (negative trion) as shown in the inset of Figure 2c. Because trions own a lower energy due to the additional Coulomb interaction from the extra charge carrier, resulting in a new PL peak at a lower energy of 1.95 eV. (II) The lattice mismatch between the boundary of WS_2_ and WSe_2_ as large as 4%^33^ can induce strain in the heterostructure. This strain can affect the band structure and, consequently, the exciton energy changed, causing the PL peak variation in the local bandgap across the heterostructure. Moreover, PL quenching in WS_2_–WSe_2_ SMS compared to individual monolayers can be significantly observed, which further demonstrates that efficient charge transfer occurs in the staggered WSe_2_–WS_2_ heterostructure (Figure S2 of the Supporting Information).^34−36^ In terms of binding energy shift in the X-ray photoelectron spectra (panels d and e of Figure 2), the results explicitly turn out the core-level energy of tungsten f orbital (blue curve in Figure 2d) and sulfur p orbital (Figure 2e) exhibits a blue shift, pointing out the electron-rich region of WS_2_ in the heterojunction structure, as shown in the inset of Figure 2c, with a reduced work function. Oppositely, the hole accumulation in WSe_2_ leads to a downward movement of Fermi level, corresponding to both binding energies a redshift for tungsten f orbital (red curve in Figure 2d) and selenium d orbital (Figure 2e).

**Figure 3:**
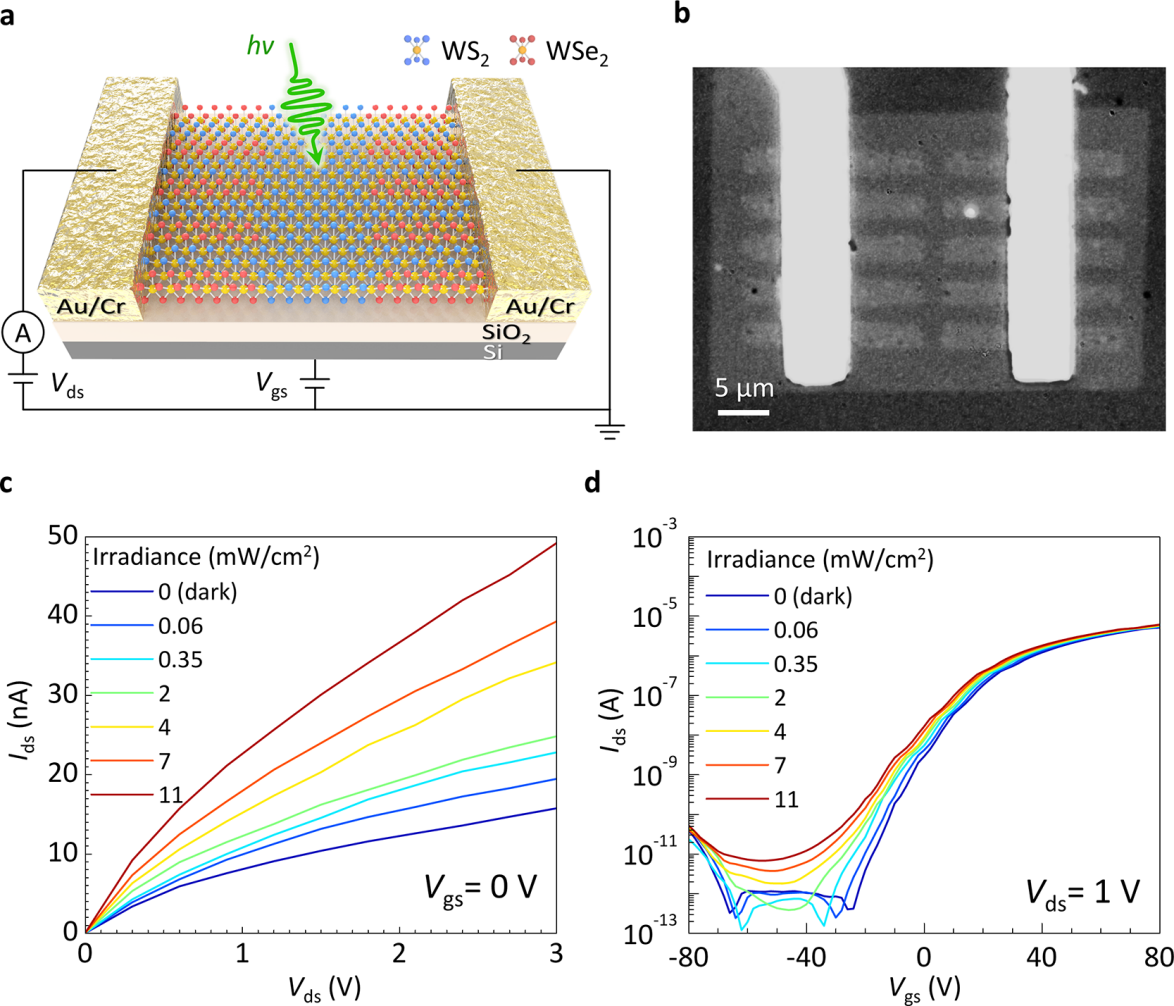
Interlayer charge transfer and scanning photoresponse of WS_2_–WSe_2_ SMS optoelectronics. (a) Device configuration of the WS_2_–WSe_2_ SMS phototransistor. (b) Optical image of the WS_2_–WSe_2_ SMS phototransistor. (c) Output characteristics at *V*_gs_ = 0 V in the dark and illumination states. (d) Transfer characteristics at *V*_ds_ = 1 V in the dark and illumination states.

Panels a and b of Figure 3 depict the WS_2_–WSe_2_ SMS phototransistor, which features a back-gate configuration. This setup includes a highly doped p-type silicon wafer, topped with a 300 nm thick SiO_2_ layer as the gate dielectric. The output characteristics of the phototransistor under varied light irradiation powers (λ = 532 nm) are depicted in Figure 3c, demonstrating a dark current of approximately 10^–8^ A at *V*_gs_ = 0 V. This level of performance is comparable to that observed in graphene-contact TMD FETs under similar conditions.^29^ Interestingly, the output characteristics of the WS_2_–WSe_2_ SMS phototransistor exhibit a unique *I*_ds_–*V*_ds_ trajectory, which is neither linear nor exponential, supposedly due to the synergistic attribution from core WS_2_ strips and longitudinal WS_2_–WSe_2_ SMS. Consequently, the depletion regions of the back-to-back p–n junctions at the WS_2_–WSe_2_ (SMS_∥_) interface can generate excess carriers that transport through the channels, particularly when exposed to light, leading to incremental flow rates. This results in a higher total current and produces exceptional *I*_ds_–*V*_ds_ output curves.

The impact of back gated configuration on the phototransistor is evident in its transfer characteristics under different lighting, as shown in Figure 3d. In dark environments, the threshold voltage (*V*_th_) sits at around −22 V, differing from pristine WS_2_ FETs.^25^ This suggests n doping in the WS_2_ channel, likely a result of charge transfer in the WS_2_/WSe_2_ type-II structure. In comparison to the pristine WS_2_ transistors, it apparently manifests the improvement of *I*_ds_–*V*_gs_ transfer curves (Figure S3 of the Supporting Information). This finding ties into the device’s field-effect mobility (μ), which is calculated using the formula, , where *l* and *w* are the channel’s length and width, and *C*_ox_ represents the gate capacitance per unit area. Remarkably, the mobility in darkness reaches 8.2 cm^2^ V^–1^ s^–1^, exceeding that of standard monolayer WS_2_ FETs.^37^ Moreover, the phototransistor exhibits a subthreshold swing (SS) and a current on/off ratio (*I*_on_/*I*_off_) of 5.5 V/dec and 1 × 10^7^, respectively, aligning with previous studies on CVD-grown TMDs (Figure S4 of the Supporting Information).

**Figure 4:**
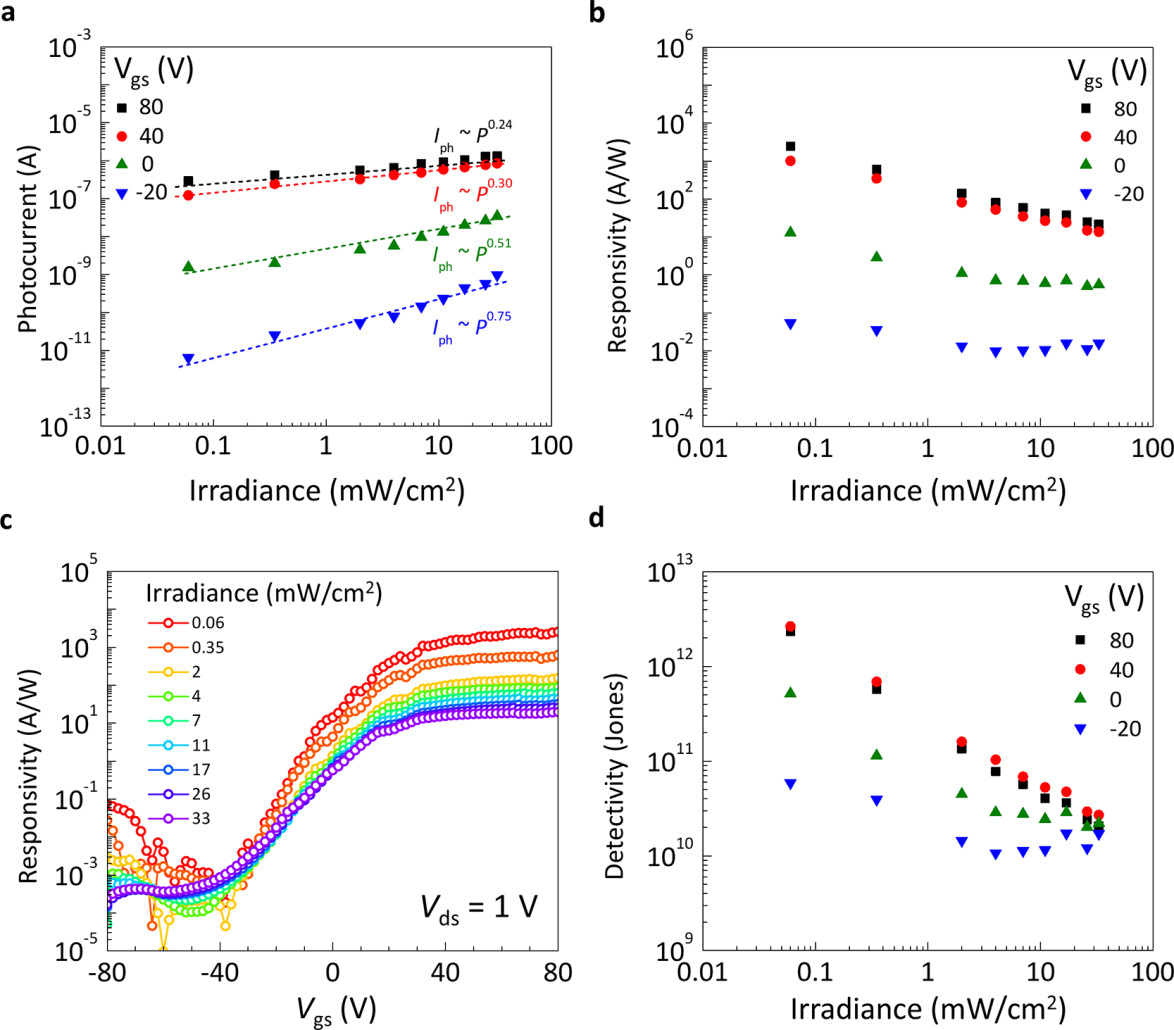
Optoelectronic characteristics of the WS_2_–WSe_2_SMS phototransistor. (a and b) Photocurrent and responsivity as a function of light power density at different gate voltage, respectively. (c) Responsivity as a function of back gate voltage at different incident power densities. (d) Specific detectivity as a function of light power density at different gate voltage.

To examine the effect of the back gate on the light detection of this device, Figure 4a points out the power density dependence of the photocurrent *I*_ph_ (*I*_ph_ = *I*_illumination_ – *I*_dark_) at different gate voltages. It reveals that the photocurrent correlates with light power *P*, described by *I*_ph_ ≈ *P*^α^. At a lower gate voltage (*V*_gs_ = −20 V), α is determined to be 0.75 across illumination power densities ranging from 0.06 to 33 mW/cm^2^. Interestingly, at a higher gate voltage (*V*_gs_ = 80 V), α decreases to 0.24. This sublinear response to optical power suggests the loss of photoexcited carriers through recombination. When the illumination intensity increases, the greater number of separated charge carriers generates an opposing electric field, thereby diminishing the built-in electric field in the depletion region.^27^ The presence of impurities at the device channel/substrate interface also plays a role in the recombination process of photogenerated carriers. At higher *V*_gs_, the occupation of interface traps within the forbidden band leads to faster recombination of photogenerated carriers.

Besides, key performance metrics such as responsivity (*R*), gain (*G*), and specific detectivity (D*) are crucial in assessing the effectiveness of the phototransistor. Responsivity, defined as *R* = *I*_ph_/*P*, varies under different light intensities and bias conditions, as depicted in Figure 4b. Impressively, at *P* = 0.06 mW/cm^2^, *V*_gs_ = 80 V, and *V*_ds_ = 1 V, *R* reaches 2.4 × 10^6^ mA/W, significantly surpassing standard monolayer TMD phototransistors.^26^ The relationship between *R* and *G* is expressed by *R* = η*Gq*/(*h*ν), where η is the external quantum efficiency, *q* is the elementary charge unit, *h* is Planck’s constant, and ν is the frequency of incident photons. With η assumed at 100%, *G* peaks at 5698, on par with heterojunction phototransistors based on III–V semiconductors.^29^ Factoring in the absorbed photons, the internal photoconductive gain (*G*_int_) is calculated as *G*_int_ = *G*/η. Given the 8% absorbance of monolayer WS_2_ under 532 nm light irradiation, *G*_int_ is estimated to be ∼7.15 × 10^4^.

To determine the sensitivity of the phototransistor, we estimate the specific detectivity D* with the formula , where *A* is the effective area of the detector, *f* is the electrical bandwidth, and *i*_n_ is the noise current, considering the device’s bandwidth, geometry, and noise. In devices based on 2D TMDs, where shot noise is typically the main noise source, D* is calculated as , where *A* is the effective area of the detector, *I*_dark_ is the measured dark current, and *q* is the elementary charge unit. Remarkably, at higher gate voltage (*V*_gs_ ≥ 40 V) in Figure 4d, the specific detectivity reaches approximately 2.6 × 10^12^ Jones, surpassing reported graphene-contacted TMD photodetectors and matching the performance of In/graphene–WS_2_–graphene photodetectors enhanced with indium adatoms.^28^ Notably, there is little variation in D* values at *V*_gs_ ≥ 40 V (Figure S5 of the Supporting Information). Similar trend can also be observed in Figure 4c with the relation between *R* and *V*_gs_ across different incident light intensities. As the gate voltage increases, it typically lowers contact barriers, which leads to more efficient extraction of photocurrent and improved photoresponse.^26^ The almost constant *R* values under light illumination, especially at higher light powers, suggest that further exploration of the metal/WS_2_ contact in the WS_2_–WSe_2_ SMS phototransistor is warranted.

**Figure 5:**
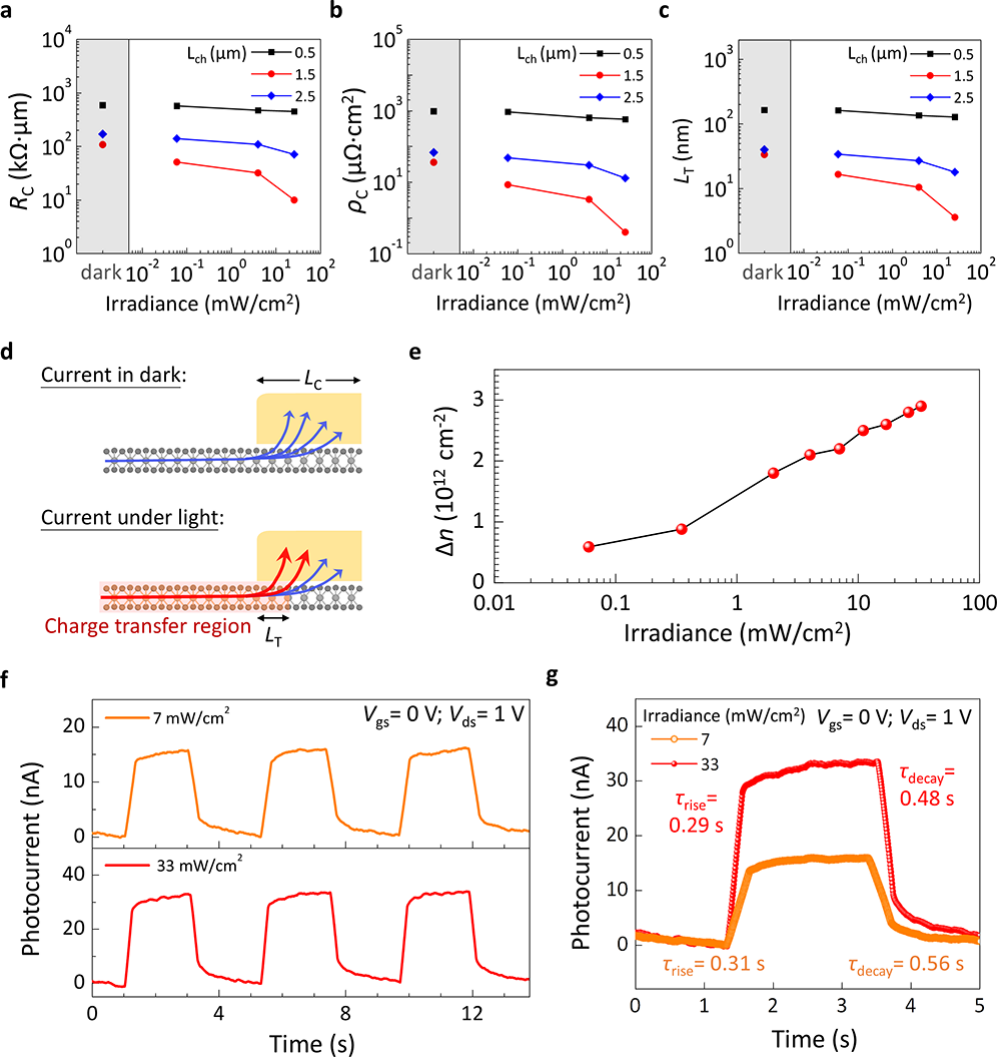
TLM results and time response of the WS_2_–WSe_2_ SMS phototransistor. (a–c) Contact resistance, specific contact resistivity, and transfer length as a function of light power density with different strip width. (d) Schematic of the decay of injected current over the transfer length at metal/WS_2_–WSe_2_ contact region before and after light illumination. (e) Excess carrier densities as a function of light power density. (f) Time-resolved photoresponse of the WS_2_–WSe_2_ SMS phototransistor at different light power density. (g) Single on/off switch at different light power density (extracted from panel f).

In our study, the transfer length method (TLM) was applied to assess the metal/WS_2_ contact. In panels a–c of Figure 5, we evaluated the contact resistance (*R*_C_), specific contact resistivity (ρ_C_), and transfer length (*L*_T_) under various lighting conditions. The TLM analysis focused on devices with different strip widths by non-destructive analysis technique, referring to the Methods section. The total resistance (*R*_total_), normalized by channel width, was determined from the *I*–*V* characteristics and channel length, following the formula , where *R*_sheet_ represents the sheet resistance (Figure S6 of the Supporting Information). *R*_C_ values obtained in the dark were 108, 170, and 585 kΩ μm for strip widths of ∼1.5, ∼2.5, and ∼0.5 μm, respectively. Notably, these values are lower than those of monolayer MoS_2_ devices and only marginally higher than those of thicker MoS_2_ devices. Under light illumination, a marked decrease in *R*_C_ was observed, particularly at higher light powers in Figure 5a.

Additionally, ρ_C_ and *L*_T_ were analyzed using equations  and , as shown in panels b and c of Figure 5. In dark conditions, both ρ_C_ and *L*_T_ were low, comparable to Ti/MoSe_2_/MoS_2_ and Cl-doped WS_2_.^38,39^ With light illumination, these values significantly decreased, suggesting enhanced charge transfer within the WS_2_–WSe_2_ SMS. This charge transfer, affected by the strip width, was more pronounced at the WS_2_/WSe_2_ interface. Interestingly, the carrier densities of photogenerated electrons and holes were higher in channels with ∼1.5 μm strip width compared to those with ∼2.5 μm. However, for ∼0.5 μm strips, recombination in depletion-dominated channels limited charge transfer, as shown in Figure 5d. To further investigate, we calculated the figure density (Δ*n*) using a parallel-plate capacitor model: Δ*n* = *C*_ox_Δ*V*_th_/*q*, with findings depicted in Figure 5e. At a power density of 33 mW/cm^2^, Δ*n* was estimated at 2.9 × 10^12^ cm^–2^, corresponding to degenerate electron doping in the 2D WS_2_ channel and a lowered contact barrier at the metal/WS_2_ interface.

Finally, to achieve the carrier dynamics under light, the time-resolved photoresponse of our phototransistor was analyzed in panels f and g of Figure 5. The device exhibited a rise time (τ_rise_) of 0.29 s and a decay time (τ_decay_) of 0.48 s with no persistent photoconductance in the off-current state. The photoconductive gain (*G*) is calculated as , agreed with experimental values, where τ_life_ is the carrier lifetime and τ_transit_ is the transit time. We can calculate the transit time using the equation , where μ is the carrier mobility and *l* is the channel length. At *V*_ds_ = 1 V and *V*_gs_ = 0 V, the mobility is measured to be 0.24 cm^2^ V^–1^ s^–1^ under the illumination power of 33 mW/cm^2^. τ_life_ and τ_transit_ are 0.48 s and 3.6 μs, resulting in a photoconductive gain of 1.3 × 10^5^. This value is in reasonable agreement with the experimental value (*G*_int_) of ∼7.15 × 10^4^, further affirming the charge transfer behavior and the impact of strip width on the WS_2_–WSe_2_ SMS phototransistor’s performance.

## Conclusion

**Figure 6:**
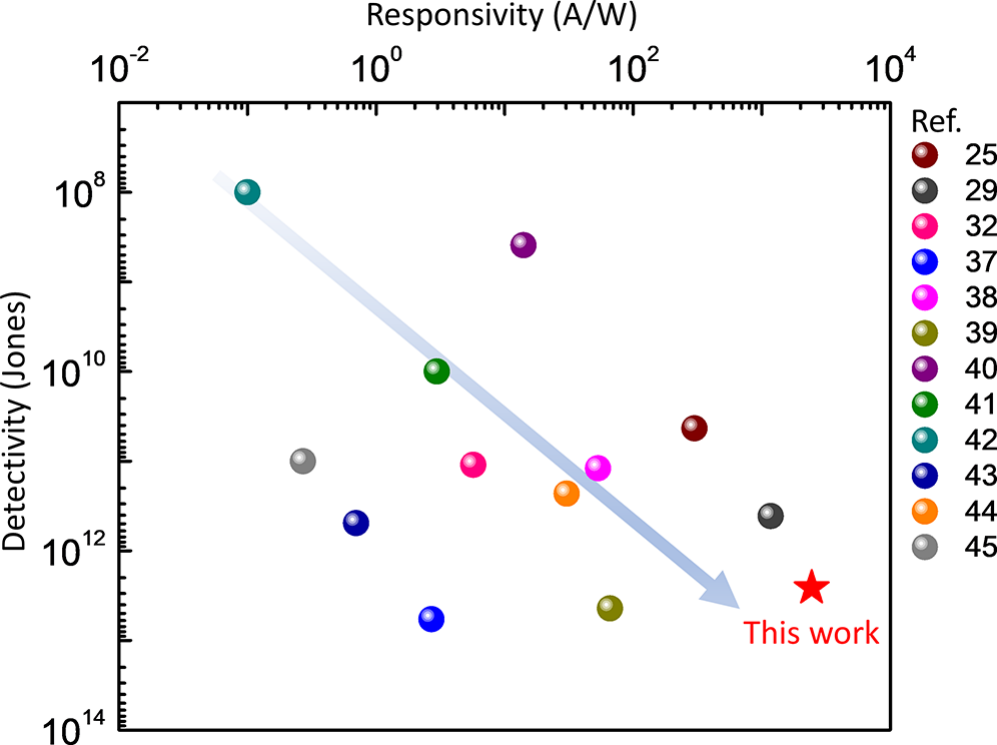
Comparison of WS_2_-based photodetectors with regard to both responsivity and detectivity.

In summary, this study successfully developed a phototransistor using van der Waals heterostructures of WS_2_ and WSe_2_. The device features sub-micrometer-sized WS_2_ and WSe_2_ strips that exhibit a type-II staggered band alignment. This alignment results in significant electron doping (∼1 × 10^12^ cm^–1^) upon light illumination. Consequently, the phototransistor demonstrates a specific detectivity of ∼2.6 × 10^12^ Jones and a remarkably high responsivity of up to 2.4 × 10^6^ mA/W with an internal photoconductive gain of ∼7.15 × 10^4^. This positions it as a leading contender in both responsivity and specific detectivity compared to other WS_2_-based photodetectors^25,29,32,40−48^ (Figure 6). Furthermore, a notable enhancement in contact resistance was observed, attributable to S1 electron doping. This improvement facilitates efficient photogenerated carrier injection through the metal/TMD contact, underscoring the device’s advanced capabilities in photodetection applications.

## Methods

### Lateral Growth of WS_2_–WSe_2_ Heterostructure

We grow the single-layer WS_2_ film on a sapphire substrate using a low-pressure chemical vapor deposition (LPCVD) system.

Tungsten oxide (WO_3_, 99.8% purity) and sulfur (S, 99.98% purity) powders purchased from Sigma-Aldrich were utilized as solid precursors. During the LPCVD process, the growth temperature and pressure were set at 850 °C and 15 Torr, respectively, with steady argon and hydrogen gas flow rates of 200 and 20 sccm. Next, to pattern the single-layer WS_2_ film into periodic arrays, we used electron-beam lithography first, and then a partial etching of WS_2_ was performed using oxygen plasma. Thereafter, the patterned single-layer WS_2_ film was placed into another LPCVD system to conduct the lateral growth of WSe_2_. The experimental setup and procedure are similar to those for the single-layer WS_2_ film mentioned in the previous paragraph, involving the usage of WO_3_ powder as a metal precursor. To evade the risk of thermally induced defect states created in the patterned single-layer WS_2_ film, the growth temperature for WSe_2_ was set at 750 °C for 7 min, which was under the growth temperature of WS_2_. When the growth was finished, the LPCVD system was cooled to room temperature with argon gas as a protective mean. The schematic process flow can be referred to as shown in Figure S1 of the Supporting Information.

### Device Fabrication

In the process of fabricating the WS_2_–WSe_2_ SMS phototransistor, initially, a thin layer of polypropylene carbonate (PPC) solution equipped by dissolving the PPC in chloroform was spin-coated onto the sapphire substrate for transferring the WS_2_–WSe_2_ strip superstructure. Afterward, the PPC/WS_2_–WSe_2_ strip superstructure was separated from the sapphire substrate and transferred onto a 300 nm SiO_2_/P^+^–Si substrate by a wet etching process using buffered HF solution composed of a 6:1 volume ratio of 40% NH_4_F and 49% HF in water. Then, we utilized P^+^–Si with 300 nm SiO_2_ as the global back control gate and the gate dielectric, separately. To create source/drain electrodes, the WS_2_–WSe_2_ strip superstructure was defined using photolithography, followed by thermal evaporation for Cr/Au (0.5/80 nm). To pursue the device isolation, the unnecessary area around the WS_2_–WSe_2_ strip superstructure was removed using electron-beam lithography and oxygen plasma etching. The process flow can be referred to as shown in Figure S1 of the Supporting Information.

### Raman Characterization

A high-resolution micro-Raman spectrometer (DXR, Thermo Scientific) with a motorized sample stage was used to obtain the spectra. The measurement was performed at room temperature with an excitation wavelength of 532 nm. The laser spot size was focused with an objective lens of approximately 1 μm in diameter. The low-power laser (below 0.1 mW) was employed to prevent sample damage because of laser-induced heating.

### Optoelectronic Measurements

For the optoelectronic measurements, a solid-state laser with the spot size of ∼1 mm in diameter was set as the light source. Light illumination covers the whole area of the devices to ensure the accuracy of measurements. The output power is tunable from 100 μW to 80 mW. A semiconductor characterization system (Keithley 4200A-SCS) was employed as the voltage source measure unit for the two-terminal and three-terminal optoelectronic measurements. All device measurement processes are under vacuum states below 10^–1^ Torr to isolate the influence of air gas and moisture, by which relevant hysteresis can be efficiently eliminated.

## Supporting Information Available

Process for the formation of WS_2_–WSe_2_ SMS phototransistor (Figure S1), PL intensity quench for the WS_2_–WSe_2_ SMS heterojunction (Figure S2), comparison of electrical properties and photoresponse between pristine WS_2_-based transistor and WS_2_–WSe_2_ SMS phototransistor (Figure S3), output and transfer characterizations for WS_2_–WSe_2_ SMS phototransistor (Figure S4), specific detectivity for WS_2_–WSe_2_ SMS phototransistor (Figure S5), and TLM measurement for WS_2_–WSe_2_ SMS heterojunction (Figure S6) (PDF)
