# Supplementary material for: Two-Dimensional Phototransistors with van der Waals Superstructure Contacts for High-Performance Photosensing
Source: ACS Appl Mater Interfaces. 2025 Jan 13;17(4):6521–9. doi: 10.1021/acsami.4c16883 (PMC11788984; doi:10.1021/acsami.4c16883)
Supplement: Supplementary file 1 — am4c16883_si_001.pdf [file am4c16883_si_001.pdf]

Supporting Information

2D Phototransistors with van der Waals Superstructure Contacts for High-Performance Photosensing

Ming-Deng Siao,[1] Meng-Yu Tsai,[14] Ashish Chhaganlal Gandhi,[1] Yi-Chung Wu,[1] Ta Fan,[1] Li-Syuan, Hao[2], I-Ling, Li[5], Sun-Zen Chen,[3] Chang-Hua Liu,[1] Yen-Fu Lin[4], and Chao-Hui Yeh[1,2,3,5]*

[1]*Department of Electrical Engineering, National Tsing Hua University, Hsinchu 30013, Taiwan.*

[2]*Institute of Electronics Engineering, National Tsing Hua University, Hsinchu 30013, Taiwan.*

[3]*Center for Nanotechnology, Materials Science and Microsystem, National Tsing Hua University, Hsinchu 30013, Taiwan.*

[4]*Department of Physics, National Chung Hsing University, Taichung 40227, Taiwan.*

[5]*College of Semiconductor Research, National Tsing Hua University, Hsinchu 30013, Taiwan.*

* Correspondence to: chyeh@ee.nthu.edu.tw

Content of supporting information:

S1. Process for the formation of WS_2-WSe_2 SMS phototransistor

S2. PL intensity quench for the WS_2-WSe_2 SMS heterojunction

S3. Comparison of electrical properties and photoresponse between pristine WS_2-based transistor and WS_2-WSe_2 SMS phototransistor

S4. Output and transfer characterizations for WS_2-WSe_2 SMS phototransistor

S5. Specific detectivity for WS_2-WSe_2 SMS phototransistor

S6. TLM measurement for WS_2-WSe_2 SMS heterojunction

S1. Process for the formation of WS_2-WSe_2 SMS phototransistor

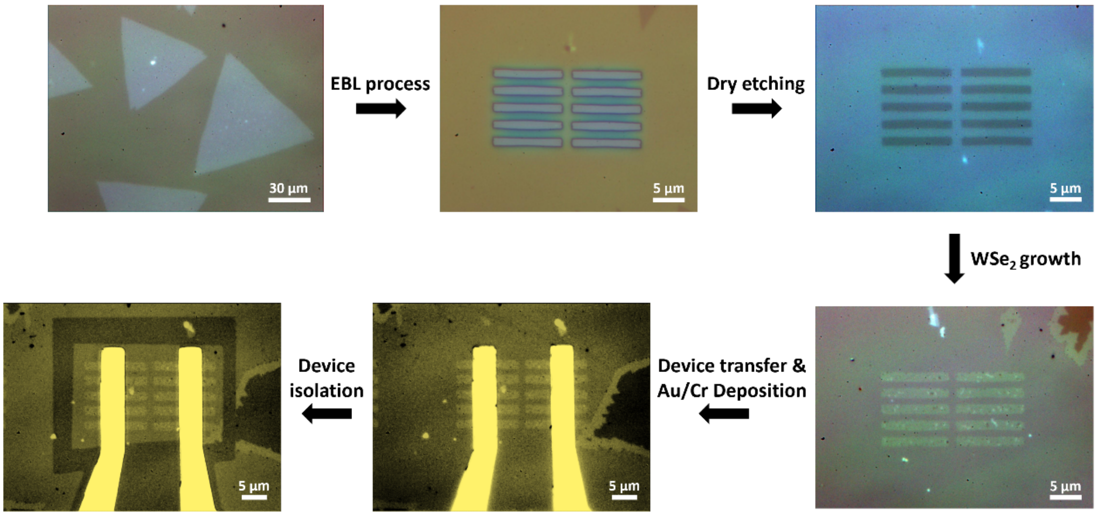

Figure S1 | The process of device fabrication is presented by OM images. The large-sized monolayer WS_2 crystals synthesized by CVD is patterned by EBL and etched by oxygen plasma for selective etching. The WSe_2 SMSs are grown by CVD from the edges of patterned WS_2, forming the WS_2-WSe_2 heterojunction. The heterojunction is then transferred onto the SiO_2/P^+Si substrate for back-gate fabrication, and source/drain metal pads (Cr/Au) are created using photolithography and thermally evaporation. The unnecessary area around the heterojunction is removed for device isolation using EBL and oxygen plasma etching.

S2. PL intensity quench for the WS_2-WSe_2 SMS heterojunction

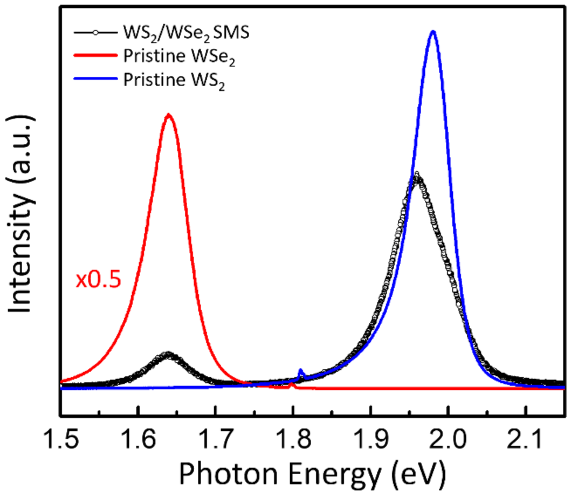

Figure S2 | Comparison of PL spectra between pristine WS_2, pristine WSe_2 and WS_2--WSe_2 SMS heterojunction, respectively. The peak intensities of both WS_2 and WSe_2 are significantly quenched compared to pristine WS_2 and WSe_2, attributing to charge transfer across the interface.

S3. Comparison of electrical properties and photoresponse between pristine WS_2-based transistor and WS_2-WSe_2 SMS phototransistor

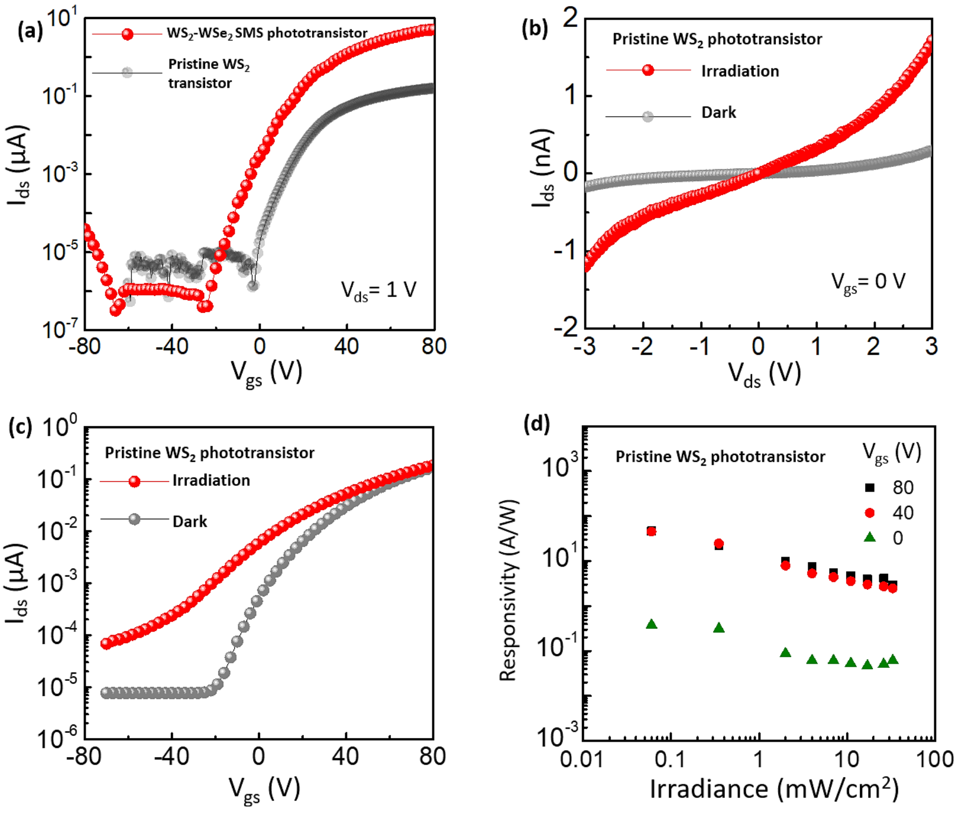

Figure S3 | (a) I_{ds}-V_{gs} transfer curves of the pristine WS_2 transistor and WS_2-WSe_2 SMS phototransistor at V_{ds} = 1 V (L_{ch} = 10 μm). (b) I_{ds}-V_{ds} output curves of the pristine WS_2 transistor in the dark and under light irradiation of 0.3 mW/cm^2. (c) The measured I_{ds}-V_{gs} curves as a function of gate voltage for the pristine WS_2 transistor in the dark and under light irradiation of 0.3 mW/cm^2. (d) Responsivity of pristine WS_2 transistor as a function of incident light power densities at different applied back gate voltages.

S4. Output and transfer characterizations for WS_2-WSe_2 SMS phototransistor

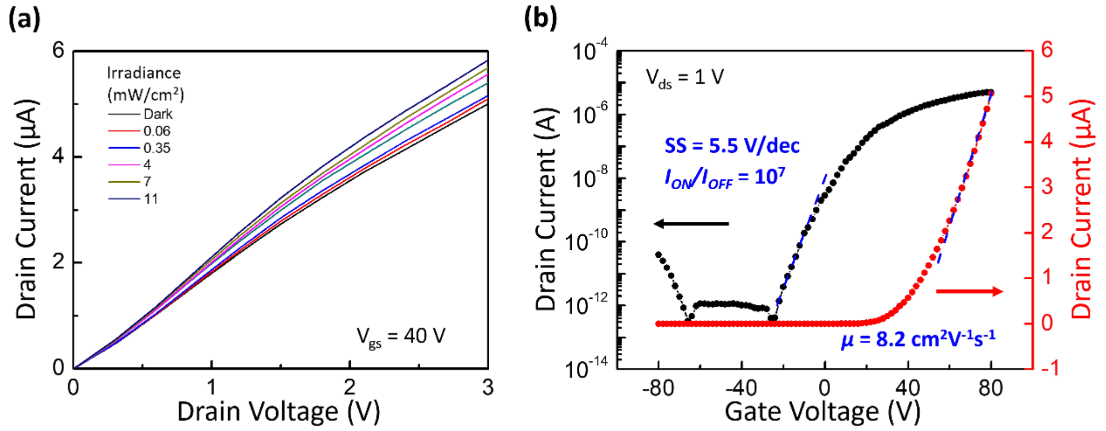

Figure S4 | (a) I_{ds}-V_{ds} curves of the WS_2-WSe_2 SMS phototransistor at V_{gs} = 40 V in the dark and illuminated states. (b) I_{ds}-V_{gs} curves of the WS_2-WSe_2 SMS phototransistor on linear scale (red) and logarithmic scale (black) in the dark. The field-effect mobility (μ), subthreshold swing (SS) and current on/off ratio I_{on}/I_{off} can reach 8.2 $cm^2V^{-1}s^{-1}$, 5.5 V/dec and 1 x 10^7, respectively.

S5. Specific detectivity for WS_2-WSe_2 SMS phototransistor

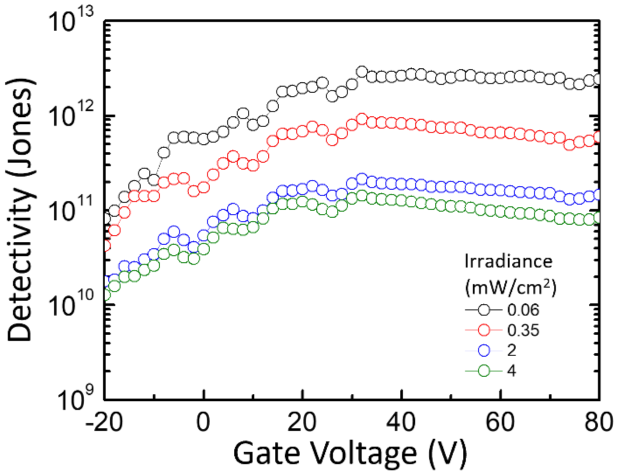

Figure S5│ Specific detectivity (*D**) as a function of back gate voltage at different incident power densities. The *D** value nearly saturates as $V_{gs} \geq 40$ V.

S6. TLM measurement for WS_2-WSe_2 SMS heterojunction

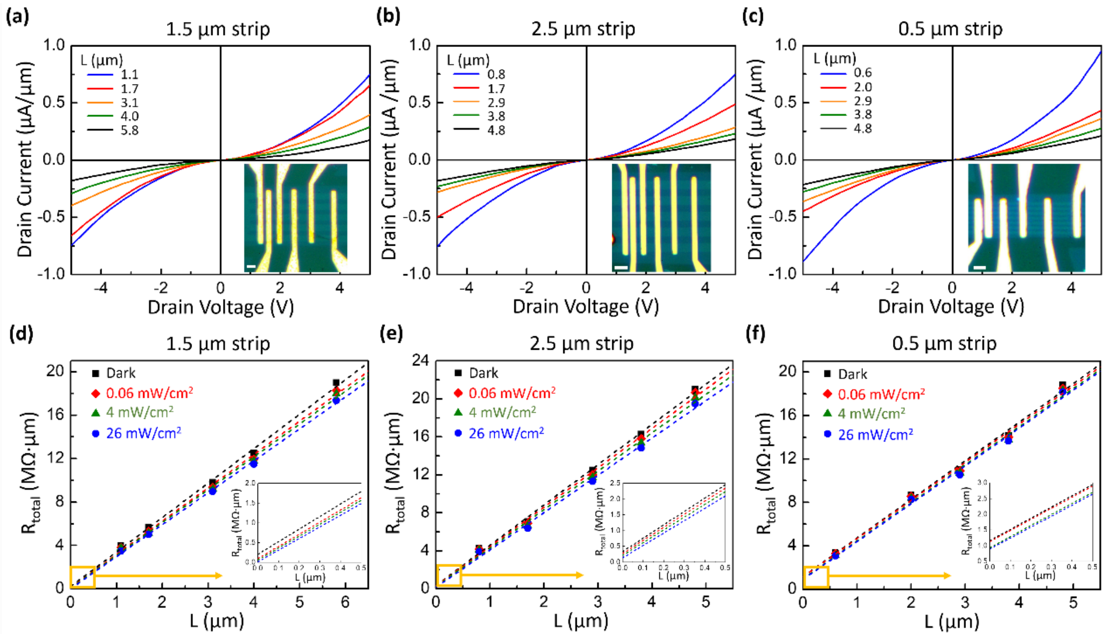

Figure S6 | (a)-(c) show the output characteristics of the WS_2-WSe_2 SMS heterojunction at V_{gs} = 0 V. (d)-(f) show the total resistance as a function of channel length. The inset shows the extraction of contact resistance by y-intercept.
